# A Comparison Between Three Types of Scaffolds for Pulp Regeneration: A Histological Study on Dogs

**DOI:** 10.1002/cre2.70031

**Published:** 2024-10-23

**Authors:** Aliaa Alshahhoud, Mhd. Salem Rikab, Nizar Issa, Ahmad Manadili, Yasser Alsayed Tolaibah

**Affiliations:** ^1^ Department of Endodontics and Operative Dentistry, Faculty of Dentistry Damascus University Damascus Syria; ^2^ Department of Biology, Faculty of Science Damascus University Damascus Syria; ^3^ Department of Pathology, Faculty of Dentistry Damascus University Damascus Syria; ^4^ Department of Pediatric Dentistry, Faculty of Dentistry Damascus University Damascus Syria

**Keywords:** chitosan, regenerative endodontics, root canal therapy, scaffolds

## Abstract

**Objectives:**

This study aims to compare the application of three types of normal scaffolds—native chitosan, enzymatically modified chitosan, and blood clot (BC)—on pulp regeneration in the teeth of experimental dogs through histological examination, to determine the quantity and type of new tissues formed within the root canal.

**Materials and Methods:**

The research sample consisted of 32 root canals from 20 premolars of two male local experimental dogs. The sample was randomly divided into a control group, in which no intervention was performed on the teeth, and three experimental groups based on the type of scaffold used: the BC group, the native chitosan combined with BC (NCS + BC) group, and the enzymatically modified chitosan combined with BC (EMCS + BC) group. Mechanical and chemical cleaning of the canals was performed, followed by the application of the studied scaffolds within the root canals. After 3 months, the teeth were extracted and prepared for histological study, where two variables were studied: the percentage of total vital tissue (soft and hard; VT%) and the percentage of soft vital tissue only (ST%). A one‐way ANOVA and Bonferroni tests were used to determine significant differences between the groups at a 95% confidence level.

**Results:**

The VT% values were significantly higher in the EMCS + BC group compared to both the NCS + BC and BC groups. The ST% values were also significantly higher in the EMCS + BC group compared to the BC group. However, no significant differences in ST% values were observed between the NCS + BC group and either the BC or EMCS + BC groups.

**Conclusions:**

Within the limitations of this study, we conclude that the application of enzymatically modified chitosan scaffolds combined with BC yields superior results in pulp regeneration, which contributes to the formation of pulp‐like tissue and cells resembling odontoblasts, as well as apex closure with tissue resembling bone tissue.

## 1 Introduction

Over the past few decades, there has been significant interest in therapeutic approaches focused on regenerating functional dental pulp within the devitalized endodontic space (Rosa et al. 2013). This has led to the emergence of regenerative endodontic therapy as a contemporary treatment alternative for necrotic immature teeth (Bose, Nummikoski, and Hargreaves 2009). Regenerative endodontic procedures (REPs) refer to biologically based procedures that aim to restore damaged structures such as dentin, root and periapical tissues, as well as cells within the pulp–dentin complex (Murray, Garcia‐Godoy, and Hargreaves 2007; Law 2013; Ong et al. 2020). Achieving optimal results in REPs depends on four important principles: (1) using a disinfection protocol that effectively disinfects the root canal to create a microenvironment conducive to stem cell proliferation and differentiation; (2) using a biocompatible scaffold; (3) utilizing growth factors to promote proliferation and differentiation; and (4) applying chemotactic factors to facilitate the organized migration of stem cells into the root canal area (Kim et al. 2010).

The effective process of dental regeneration involves the proliferation of potent stem cells residing in dental soft tissues, followed by their differentiation into specific secretory cells essential for lost tissue replacement (Sloan and Waddington 2009).

A crucial aspect of achieving success in tissue regeneration involves the use of biomaterials as scaffolds, designed as three‐dimensional (3D) structures that support cell growth and differentiation, promote cell adhesion, and facilitate migration (Nakashima and Akamine 2005).

Natural materials derived from animals or plants (e.g., collagen, gelatin, alginate, cellulose, chitosan, fibrin, and hyaluronan), as well as synthetic materials such as bioactive ceramics and a wide range of synthetic polymers, have been used for the fabrication of the scaffolds (Chan and Leong 2008). Furthermore, several recent studies indicate that chitosan‐based scaffolds are valuable biomaterials with a wide range of biomedical applications (Fakhri et al. 2020).

Chitosan is a copolymer of glucosamine and *N*‐acetylglucosamine units linked by a 1–4 glucosidic bond, and it is a deacetylated derivative of chitin (d'Aquino et al. 2009). This polysaccharide possesses several characteristics that make it highly suitable for biomedical applications, including anticholesterolemic and antimicrobial properties, biodegradability, biocompatibility, noncarcinogenicity, hemostatic potential, fungistaticity, high affinity for proteins, and the ability to promote cell adhesion, proliferation, and differentiation (Kim et al. 2008). The use of chitosan in the form of stem cell‐seeded tubes/scaffolds has shown success in promoting the adhesion, proliferation, and differentiation of stem cells, thereby facilitating the replacement of lost neurons at injury sites. Specifically, chitosan‐based 3D porous scaffolds can provide a favorable and conducive microenvironment for the attachment, survival, and neural differentiation of dental pulp stem cells (Feng et al. 2014).

Chitosan‐based scaffolds have enhanced the proliferation, migration, and odontoblastic differentiation of dental pulp stem cells and mesenchymal stem cells (MSCs) both in vivo and in vitro (Albuquerque et al. 2014; Ducret et al. 2019).

Nevertheless, the inferior mechanical properties and high swelling ability of chitosan can lead to easy deformation, which limits its use in biomedical applications (Bettini et al. 2008). To overcome these drawbacks and improve cell adhesion, several studies have reported on the chemical modification of chitosan to enhance its physicochemical and mechanical properties, such as its hydrophobic character and surface topography (Hozumi et al. 2010). Recently, enzymatic tools of chitosan modification have been demonstrated as an attractive alternative to toxic, environmentally unfriendly, and nonspecific chemical approaches (Aljawish et al. 2012, 2015).

Enzymatically modified chitosan with phenol is nontoxic to MSC cells and exhibits much higher antioxidant activity compared to novel chitosan (Aljawish et al. 2015). However, the effects of enzymatically modified chitosan with phenol compounds on dental pulp regeneration have not yet been documented. The purpose of this study is to assess and histologically analyze pulp regeneration when enzymatically modified chitosan scaffolds are applied to immature dog teeth and to compare the results with other scaffold types.

This research is one of the first histological studies that aimed to evaluate the outcome of pulp regeneration using enzymatically modified chitosan scaffolds in comparison with blood clots (BC) and native chitosan scaffolds in immature dog teeth.

## 2 Methods

### 2.1 Study Design

This study has been reported in line with the ARRIVE guidelines 2.0 for reporting animal research. It is an experimental, controlled histological study designed to compare the application of different scaffolds (BC, native chitosan, and enzymatically modified chitosan) in pulp regeneration, as well as the quantity and type of newly formed tissues. This will be achieved by conducting histological examinations using a light microscope.

### 2.2 Ethical Approval

Ethical approval for this study was obtained from the Scientific Research Ethics Committee at the Faculty of Dentistry at Damascus University (UDDS‐1189‐25092018/SRC‐74).

### 2.3 Sample Size Calculation

The sample size was determined using G*Power software (version 3.1.9.7, Düsseldorf, Germany), based on a previous study conducted by Zhu et al. (2013), and calculated with a type I error (*α*) of 0.05, a power (1 − *β*) of 0.95, and a possible large effect size (effect size = 2.64).

### 2.4 Sample and Procedures

#### 2.4.1 Inclusion Criteria

The research sample consisted of 32 immature roots from 20 upper and lower premolars, with intact crowns and roots after being confirmed by radiographic examination. These were obtained from two healthy male local experimental dogs, approximately 7–9 months old and weighing 30–35 kg, that had not undergone any previous procedures. The dogs were housed in the animal farms at the Faculty of Agriculture, Damascus University, Syria, in separate kennels under typical ambient conditions (25°C, and a 12‐h light/dark cycle) with twice daily maintenance meals and free access to water.

The dogs were given the five‐in‐one vaccine (DHPPI), followed by a booster dose after 21 days, and then they were given the rabies vaccine.

The premolars were selected as dogs have four premolars in each half of the jaw, the first premolar has a single canal, whereas the second, third, and fourth premolars have two canals. A total of 32 root canals (from 20 premolars) were distributed into four groups: three experimental groups (BC, native chitosan, and enzymatically modified chitosan) consisting of 24 root canals (12 bi‐canal premolars) and a control group (CG) with eight single‐canal premolars.

#### 2.4.2 Randomization and Blinding

The study followed a split‐mouth design in which all study groups were represented in each dog and randomly distributed among the quadrants of the mouth. A random sequence was generated using the website www.random.org to allocate the teeth into three groups. Six sheets of paper, numbered from 1 to 6 for each dog, were individually placed in opaque envelopes. An assistant not involved in the research selected an envelope before the start of the experimental procedures. The number in the envelope determined the regenerative protocol the teeth would undergo.

Number coding was done before the experimental procedures began, and the results were measured and analyzed later. The numerical codes were known by an assistant who was not involved in the research, whereas the outcomes assessors remained blinded to the group allocations.

#### 2.4.3 Teeth Preparation

All procedures were performed at the Veterinary Medicine Clinics of the Faculty of Agriculture, Damascus University, Syria, and were conducted under general anesthesia. Each dog was administrated with Xylazine (Jawa Pharmaceuticals Corporation, Haryana, India) intravenously at a dose of 0.1 mg/kg of the dogs's weight, and an intramuscular injection of ketamine (Jawa Pharmaceuticals Corporation, Haryana, India) at a dose of 0.4 mg/kg of the dog's weight. The oral cavity was disinfected with povidone solution (AL MOURAD Corporation, Aleppo, Syria), and the immaturity of the apices was confirmed by periapical radiographs. Local anesthesia (3% mepivacaine without a vasoconstrictor, Avocaine Dental 3% from Avenzor Corporation, Damascus, Syria) was administered only for the teeth in the experimental groups. Then, the dog's teeth were wiped with gauze moistened with 5.25% sodium hypochlorite (Shahbaclor, Shahbadend Corporation, Aleppo, Syria) and isolated with cotton pallets.

An oval‐shaped access cavity was prepared in premolars with two canals using a sterilized round bur (Lusterdent Corporation, Zhengzhou, Henan, China) at high speed under a copious water spray. The working length was estimated from periapical radiographs (Saoud et al. 2015; Forghani et al. 2017), and patency was achieved using a #15 K‐file (Mani Corporation, Tochigi, Japan). After removing the pulp tissue, minimal mechanical instrumentation was performed by gently brushing the canal walls circumferentially with #80 K‐files and H‐files (Mani Corporation, Tochigi, Japan) (Lin et al. 2014).

Subsequently, heavy chemical debridement was performed using 2.5% sodium hypochlorite (Shahbaclor, Shahbadend Corporation, Aleppo, Syria) at a rate of 20 mL per canal to remove all remaining pulp tissue using special irrigant tips with side openings (Endo‐Top, FANTA‐Dental Corporation, Shanghai, China). The canals were dried with absorbent paper points (Metabiomed Corporation, Chungcheongbuk, Korea), then washed with a 17% EDTA solution (MD.cleanser, Metabiomed Corporation, Chungcheongbuk, Korea) at a rate of 10 mL per canal and left for 1 min within the canals. Finally, the canals were rinsed with 20 mL of physiological saline solution per canal (DIMAS Corporation, Damascus, Syria) (AAE 2016).

After thoroughly drying the canal with paper points, the studied scaffolds were applied according to each study group as follows:

#### 2.4.4 The CG

This group consisted of eight first premolars with a single canal. In this group, the teeth were left without any interference to allow for the natural development of the teeth roots, serving as a baseline for comparison with the other groups.

#### 2.4.5 BC Scaffold Group

This group consists of four premolars, each with two canals (a total of eight root canals) in which bleeding was induced inside the root canal by gentle over instrumentation using a #25 k‐file. It was gently twisted two to three times clockwise and counterclockwise.

Once clear bleeding was observed in the canal, reaching the level of the cementoenamel junction, a small cotton pellet soaked in saline was placed in the coronal third of the canal for 10 min to allow the BC to form (Shah and Logani 2012).

#### 2.4.6 Native Chitosan Scaffold Group (NCS** + **BC)

This group also consists of four premolars, each with two canals (a total of eight root canals). Bleeding was induced inside the root canal in the same manner as in the BC group. Afterward, native chitosan (Sigma Aldrich Corporation, St. Louis, Missouri, USA) was applied within the root canal by inserting the needle tip halfway into the root canal and slowly injecting it while gradually withdrawing the needle until the canal was filled. The chitosan is then mixed with the BC using a #25 k‐file until a homogenous mixture is obtained (AlHowaish et al. 2022).

#### 2.4.7 Enzymatically Modified Chitosan Scaffold Group (EMCS** + **BC)

This group consists of four premolars, each with two canals (a total of eight root canals) in which bleeding was induced inside the root canal in the same manner as in the BC group. Subsequently, enzymatically modified chitosan which has been modified in the Department of Biology, Faculty of Science, University of Damascus, is injected and mixed with the BC using #25 k‐file until they were homogenous (Verma et al. 2017, AlHowaish et al. 2022).

#### 2.4.8 Modifying Chitosan

Native high molecular weight chitosan (310,000–375,000 kDa) with a degree of deacetylation greater than 95% was used from Sigma Aldrich Corporation, St. Louis, Missouri, USA. The chitosan was purified of impurities to provide pure chitosan. A chitosan solution was prepared at a concentration of 1% V/W by dissolving chitosan powder in acetic acid at a concentration of 1% V/V with continuous stirring for 24 h at a temperature of 25°.

The chitosan was then purified using Unisart micron membranes (SARTORIUS Corporation, Gottingen, Germany) with a porosity of 0.22 µm under vacuum. The acidity of the purified chitosan was adjusted using sodium aqueous solution NaOH (N4) to a pH of 8. The chitosan was then washed with sterile water, dried using a special desiccator, ground, and preserved at a heat of 4°C for modification.

Chitosan was modified through chemical grafting between the NH2 amino groups of chitosan and the enzymatic oxidation products of catechin (Sigma Aldrich Corporation, St. Louis, Missouri, USA), which were obtained by oxidizing catechin with an oxidation reaction catalyzed by the laccase enzyme Suberase (Sigma Aldrich Corporation, St. Louis, Missouri, USA), which is a fungal enzyme extracted from the fungus Trametes Versicolor. The grafting process was carried out in an aqueous medium at a temperature of 30° under atmospheric conditions, where 1 g of chitosan particles was mixed with 5 mM of catechin and 45 mL of phosphate buffer in the processor. The reaction was initiated by adding 0.13 mL of the laccase enzyme Suberase and placed under continuous stirring for 4 h.

**Figure 1:**
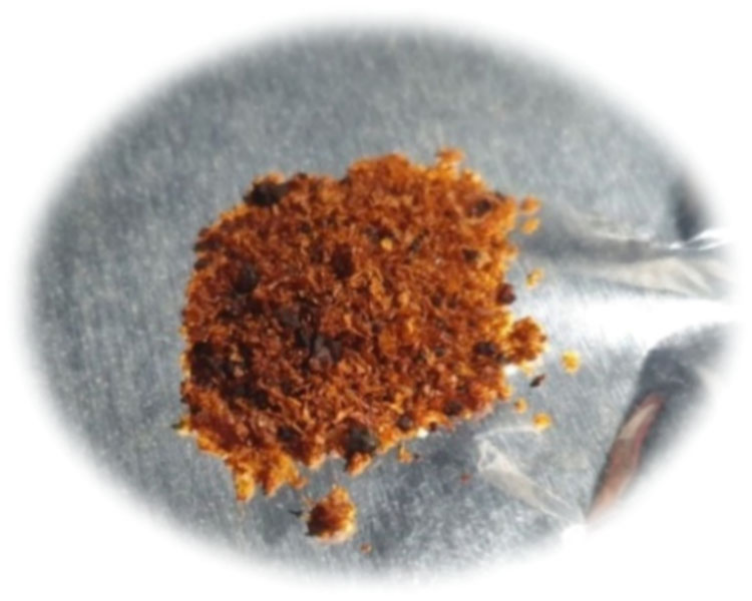
Enzymatically modified chitosan.

The reaction was then stopped by purifying the reaction mixture through micronized membranes with a porosity (0.22 µm) under vacuum. The resulting chitosan was then thoroughly washed with phosphate buffer, methanol, ethanol, and finally an acetone solution to remove all catechin and enzyme molecules from the modified chitosan. In addition, the resulting chitosan was chemically treated with an HCL/ethanol solution (50:50) and KOH/ethanol solution (50:50) at room temperature until color stability was achieved (Figure 1; Aljawish et al. 2012).

#### 2.4.9 Coronal Seal Procedures

**Figure 2:**
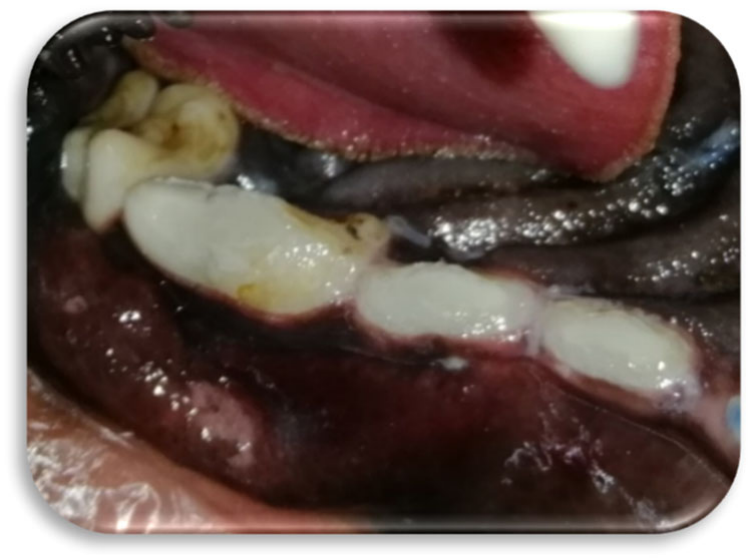
Treating and reducing the cusps of the teeth to be studied in the experimental dogs.

After applying the studied scaffolds to the experimental groups, white MTA material (MTA Cem, NEXOBIO Corporation, Chungcheongbuk, Korea) was applied. It was mixed with distilled water according to the manufacturer's instructions and applied using the MAP system (Medesy Corporation, Maniago, Italy) within the root canal orifices (Saoud et al. 2015). A layer of Glass Ionomer cement (FujiCEM, GC Corporation, Tokyo, Japan) was applied to the MTA material, and it was left to set for 5 min (Forghani et al. 2017). The teeth were then restored with a composite resin material (Tetric N‐Ceram, Ivoclar Vivadent Corporation, Schaan, Liechtenstein) (Zhu et al. 2012). Afterward, the tooth's occlusion was reduced, and all weak cusps were removed to prevent them from fracturing (Figure 2; AlHowaish et al. 2022).

At the end of the teeth preparation session, antibiotics and analgesics were administered. The animals were under postoperative care and closely monitored for any signs of discomfort or changes in eating habits. No issues were observed with their feeding.

### 2.5 Dogs Euthanization

After 3 months, the dogs were killed under general anesthesia, following the same procedure used for teeth preparation. The dogs were euthanized with an additional intravenous dose of pentobarbital (90 mg/kg of thedog's weight), and then the dogs were perfused intravenously with 10% buffered formalin at a dose of 5 mL/kg of the dog's weight (Forghani et al. 2017; Palma et al. 2017; AlHowaish et al. 2022).

After euthanization, the teeth were extracted and prepared for histological study to determine the quantity and type of newly formed tissues. This study was conducted at the Pathological Anatomy Laboratories, Faculty of Dentistry, Damascus University.

### 2.6 Histological Preparation of Teeth

The extracted teeth were fixed in 10% buffered formalin for 24 h, followed by decalcification in a 13% nitric acid solution until complete decalcification was achieved. Decalcification was confirmed by the ability to easily pierce the specimen with a sharp instrument. The samples were then washed under running water for 8 h. Then they were dehydrated using graded ethanol concentrations, cleared in xylol, and embedded in histologic paraffin. Serial longitudinal sections (4 µm thick) were then made along the mesiodistal plane (five sections per tooth). These sections were mounted on slides, deparaffinized, hydrated through descending concentrations of ethanol, and stained with hematoxylin and eosin (Saoud et al. 2015).

Each root was histologically analyzed as a separate sample unit under a light microscope (Olympus CX21, New York Microscope Company, New York, USA). The samples were then photographed and transferred to a computer for the necessary analysis.

Two observers, blinded to the study groups, analyzed all samples. The first observer (A.M.) was a professor in the Department of Pathology at the Faculty of Dentistry, Damascus University, and the second observer (M.Sh.) was a PhD student in the Department of Endodontics and Operative Dentistry, Faculty of Dentistry, Damascus University, who was well trained to analyze histological sections.

### 2.7 Outcome Measurement

Two variables were assessed from the histological images to evaluate the success of the pulpal regeneration process, the percentage of regenerated full vital tissue (both soft and hard) within the root canal, and the percentage of soft tissue only.

**Figure 3:**
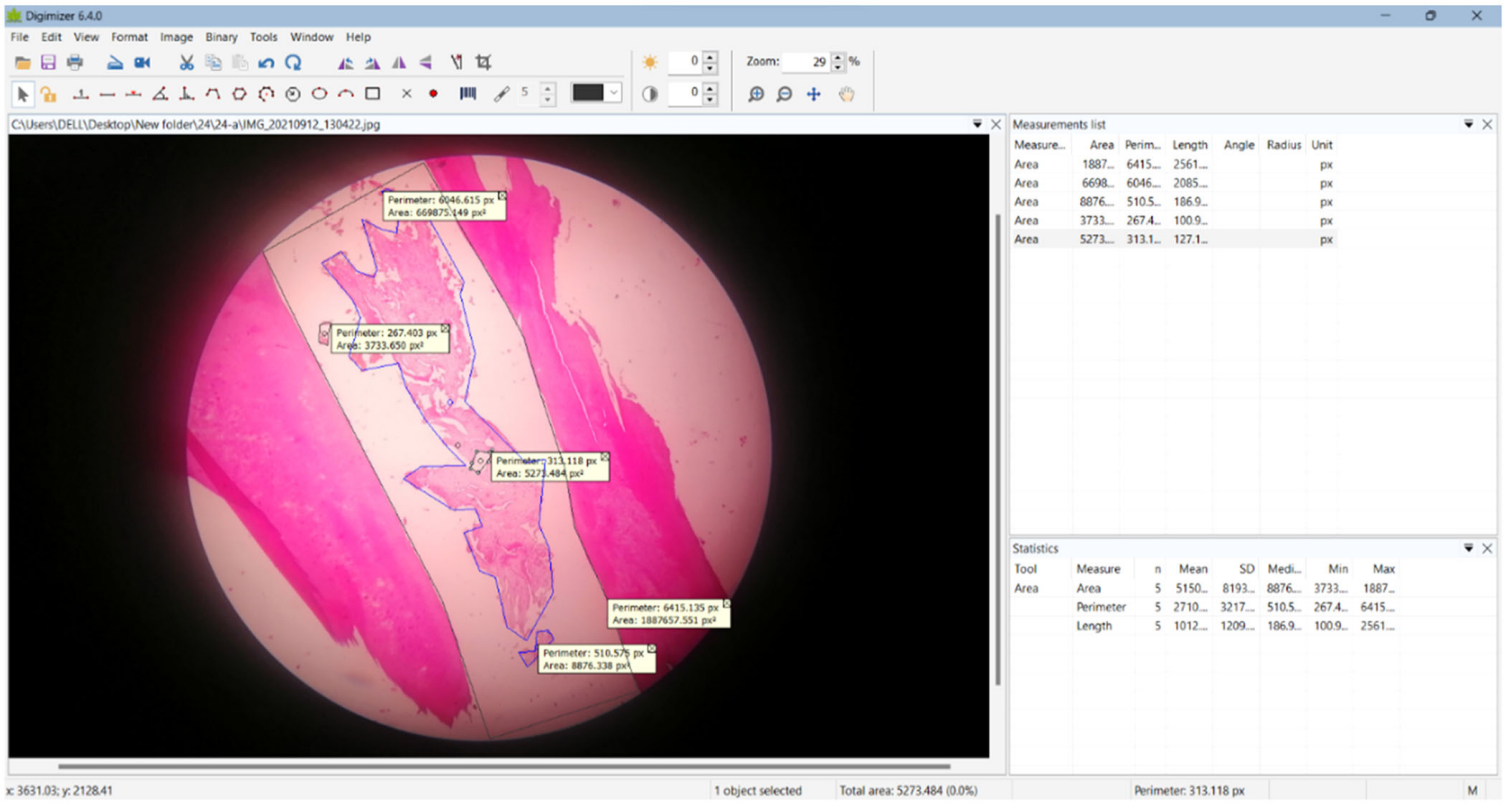
Analyzing the images and calculating the areas with the Digimizer program.

To calculate both percentages, the values of the histological images were analyzed using the Digimizer program (Zhu et al. 2013), which analyzes images and estimates areas in pixels. The program was used to calculate the area of the complete canal space bounded by the dentin walls, the area of the regenerated vital tissue (soft and hard tissues) within the pulp space, and the area of regenerated soft tissue only (Figure 3).

Then, the percentage of regenerated vital tissue was calculated using the following equation:

PercentageofFullRegeneratedVitalTissue(VT%)=(AreaofFullRegeneratedVitalTissue/AreaofCanalSpace)*100

The percentage of the regenerated soft tissue was calculated using the following equation:

PercentageofRegeneratedSoftTissue(ST%)=(AreaofRegeneratedSoftTissue/AreaofCanalSpace)*100

(Zhu et al. 2013).

In addition to these quantitative measurements, the histological analysis observed the type and location of the regenerated tissue, where the regeneration of intracanal connective tissue, intracanal mineralized tissue, odontoblast‐like cells on the dentin walls, and closure of the periapical foramen were observed.

### 2.8 Statistical Study

The analytical statistical study of the current research data has been conducted using the SPSS Software (version 13.0). The normality of data distribution was tested using the Kolmogorov–Smirnov and Shapiro–Wilk tests, which indicated a parametric (normal) distribution. Levene's test was performed to check for homogeneity of variance. According to the results, a one‐way analysis of variance (one‐way ANOVA) test was conducted to examine the significant differences in the mean percentages of vital tissue and soft tissue ratio values among the three studied groups.

When statistically significant differences were found, pairwise comparisons were performed using the Bonferroni test among the three groups to determine which of these studied groups significantly differed from the others.

## 3 Results

During the 3‐month follow‐up period of the experimental dogs, no allergic reactions, pathological changes, or behavioral changes were observed in the two experimental dogs. Additionally, there were no abscesses or fistulous openings in any of the studied teeth of the dogs, and all dental restorations remained intact. Furthermore, no teeth from the study samples were lost.

### 3.1 The CG

**Figure 4:**
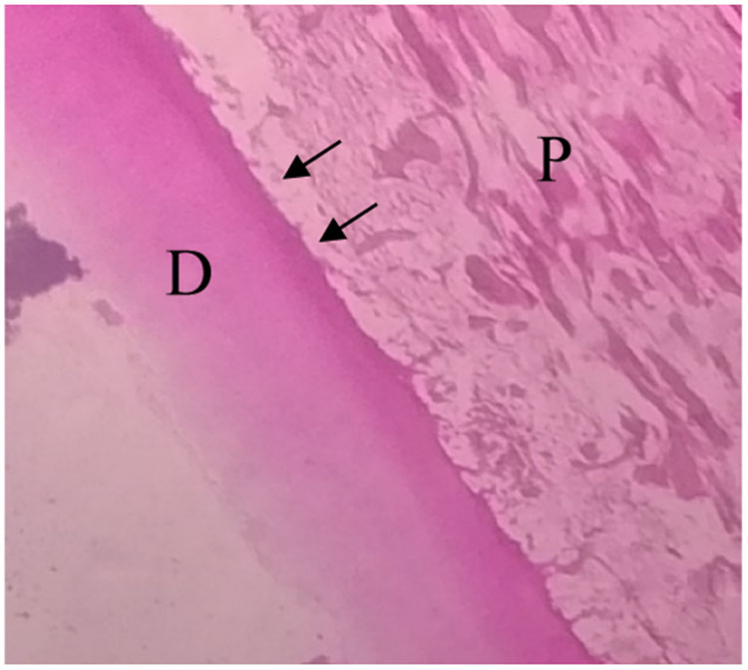
Normal pulp within the control group. D, dentin; P, normal pulp. The black arrows point to the odontoblastic cell (10×).

The results of the CG, which consisted of teeth without any interference, showed normal pulp tissue formation (Figure 4).

### 3.2 EMCS + BC

#### 3.2.1 Vital Tissue Ratio (VT%) Values

Significant differences in VT% were found among all three studied groups at a 95% confidence level (Table 1).

**Table 1:** Pairwise comparisons of vital tissue ratio (VT%) between groups.

Studied variable = vital tissue ratio (VT%)				
Group I	Group J	Mean difference (I – J)	Std. error	*p*‐value^a^
BC group	NCS + BC group	−16.74	6.36	0.047*
	EMCS + BC group	−39.18	6.36	0.000*
NCS + BC group	EMCS + BC group	−22.44	6.36	0.006*

^a^ Bonferroni test.

* Significant differences.

**Figure 5:**
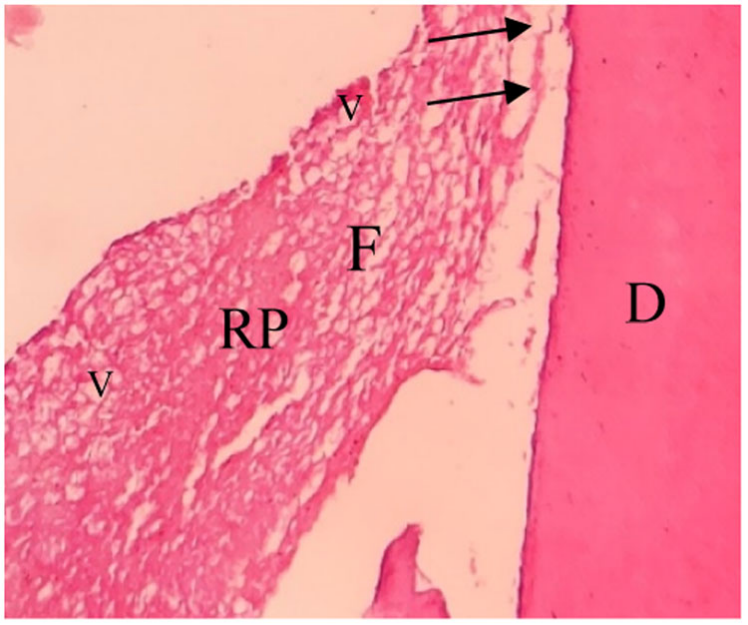
Pulp‐like tissue within the EMCS + BC group. D, dentin; F, collagen fibers; RP, renewed pulp‐like tissue; V, blood vessels. The black arrows indicate the odontoblast cells (10×).

**Figure 6:**
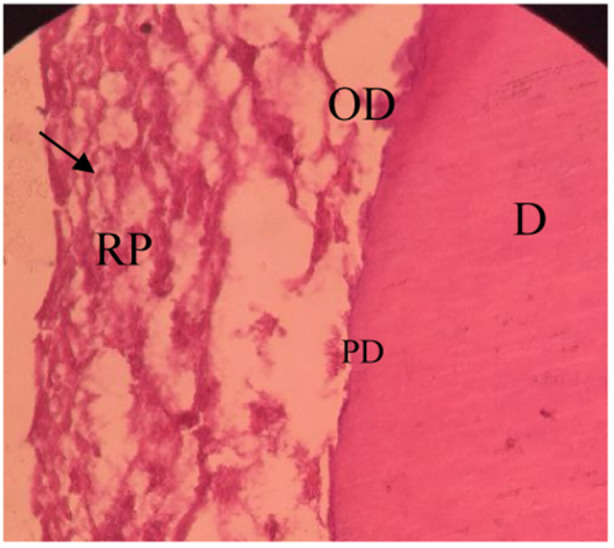
Cells similar to odontoblasts within the EMCS + BC group. D, dentin; PD, predentin; OD, cells similar to odontoblast; RP, renewed pulp‐like tissue. The black arrow indicates the odontoblast cell (40×).

**Figure 7:**
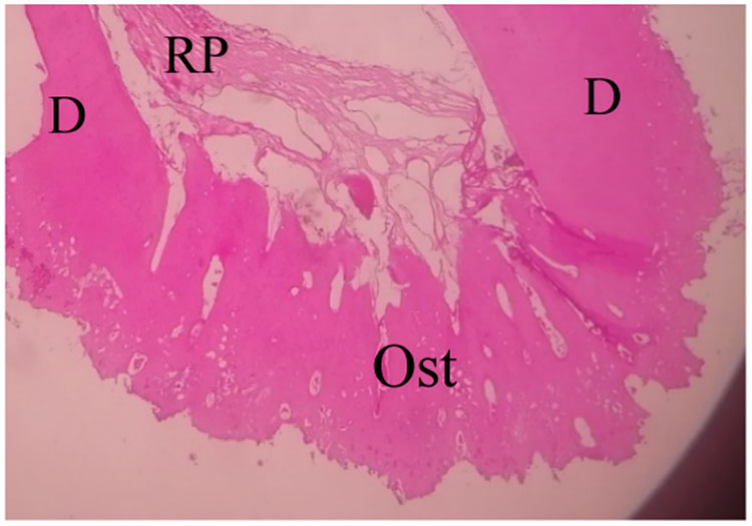
Renewed bone‐like tissue closing the apical foramen within the EMCS + BC group. D, dentin; Ost, renewed bone‐like tissue; RP, renewed pulp‐like tissue (10×).

The VT% values in the EMCS + BC group were higher than those of both NCS + BC and BC groups. The root canals in the EMCS + BC group were characterized by the formation of vital connective tissue resembling pulp tissue, which was rich in fibroblasts, fibers, and blood vessels (Figure 5), in addition to the formation of dentin‐like tissue and cells similar to odontoblasts along the dentin walls of the root canals (Figure 6) and the formation of hard tissue like bone tissue closing the apical foramen (Figure 7).

#### 3.2.2 Soft Tissue Ratio (ST%) Values

The ST% values in the EMCS + BC group were significantly higher than those in the BC group. However, there were no significant differences in the ST% values between the EMCS + BC and NCS + BC groups (Table 2).

**Table 2:** Pairwise comparisons of soft tissue ratio (ST%) between groups.

Studied variable = soft tissue ratio (ST%)				
Group I	Group J	Mean difference (I − J)	Std. error	*p*‐value^a^
BC group	NCS + BC group	−4.81	7.23	1.000
	EMCS + BC group	−22.75	7.23	0.015*
NCS + BC group	EMCS + BC group	−17.94	7.23	0.065

^a^ Bonferroni test.

* Significant differences.

### 3.3 Native Chitosan Scaffold Group (NCS + BC)

#### 3.3.1 Vital Tissue Ratio (VT%) Values

**Figure 8:**
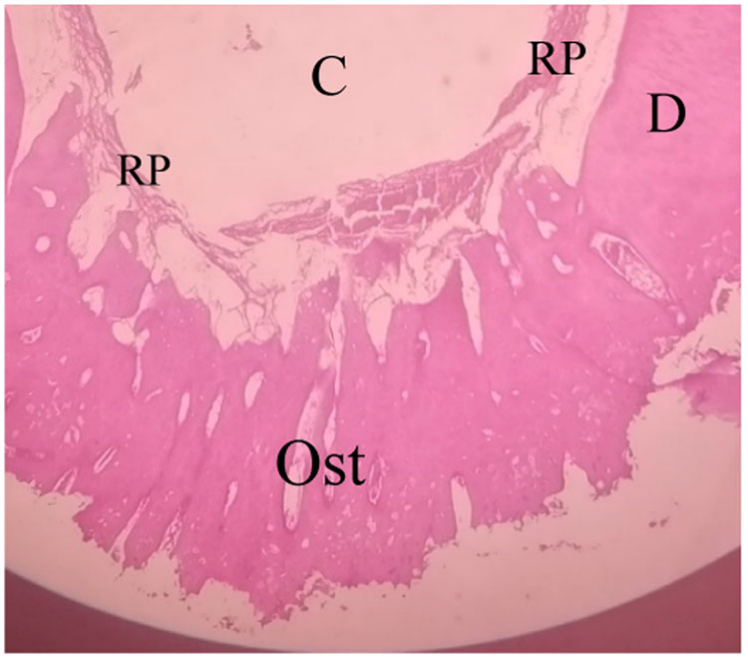
Regenerated pulp‐like tissue with regenerated bone‐like tissue that closes the periapical foramen within the NCS + BC group. C, canal space; D, dentin; Ost, renewed bone‐like tissue; RP, renewed pulp‐like tissue (10×).

The VT% values in the NCS + BC group were also higher than in the BC group in the studied sample. In this group, the formation of vital connective pulp‐like tissue was observed, including fibrous cells, fibers, and blood vessels, in addition to the formation of hard bone‐like tissue closing the periapical foramen (Figure 8).

#### 3.3.2 Soft Tissue Ratio (ST%) Values

There were no significant differences in ST% values between the NCS + BC group and both the BC and EMCS + BC groups.

### 3.4 BC Scaffold Group

#### 3.4.1 Vital Tissue Ratio (VT%) Values

The VT% values in the BC group were the lowest among the experimental groups, with significant differences observed. Tissue regeneration in this group was limited only to potential weak pulp tissue formation.

#### 3.4.2 Soft Tissue Ratio (ST%) Values

The ST% values in the BC group were significantly lower than in the EMCS + BC group, and there were no significant differences in ST% values between the BC and NCS + BC groups.

**Figure 9:**
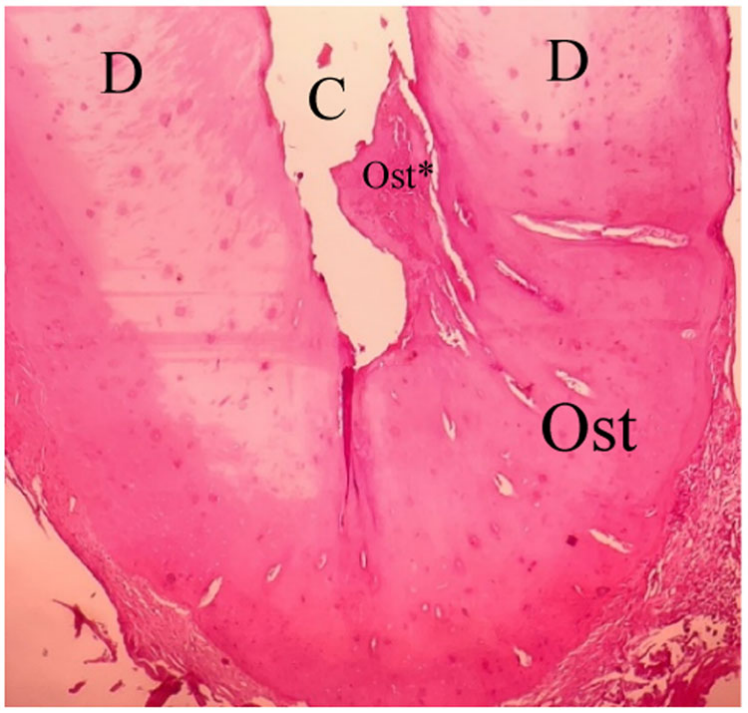
Intracanal mineralized tissue within the NCS + BC group. C, canal space; D, dentin; Ost, renewed bone‐like tissue that closes the periapical foramen; Ost*, renewed bone‐like tissue within the root canal (10×).

**Figure 10:**
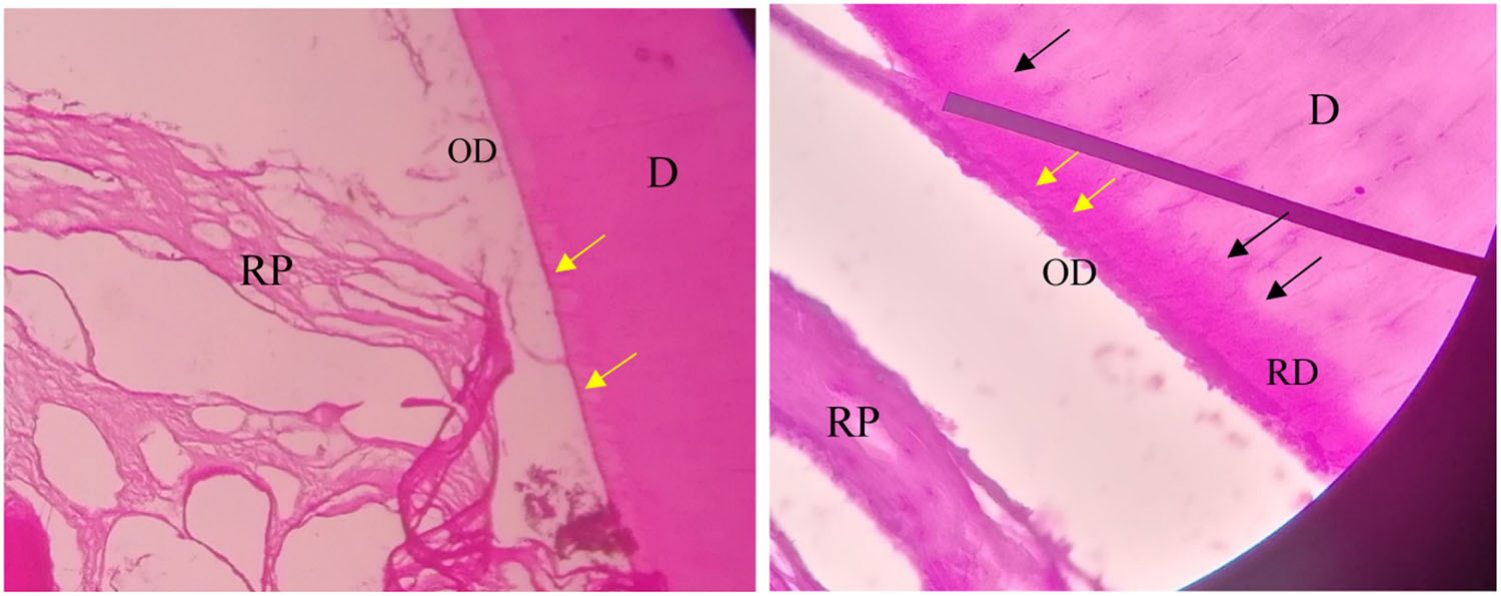
Renewed dentin‐like tissue within the EMCS + BC group. D, dentin; OD, odontoblasts‐like cells; RD, renewed dentin‐like tissue; RP, renewed pulp‐like tissue. The yellow arrows indicate the predentin layer, and the black arrows indicate the boundary between primary dentin and secondary renewed dentin (40×).

Figures 9 and 10 display both the predentin and the newly formed dentin, which appears darker than the older dentin. The images also highlight the junctions between the old and newly formed dentin, as well as the newly formed pulp‐like tissue.

## 4 Discussion

The present study evaluated the chitosan potential in pulp regeneration focusing on its distinctive properties as an antimicrobial and antifungal scaffold, along with its biocompatibility and biodegradability, which support cell growth and proliferation (Ravi Kumar 2000).

Regeneration endodontic therapy has been a research subject for decades, both in animals and humans, with a wide range of protocols, materials, and success criteria being evaluated.

Cell transplantation has been heavily relied upon in these experiments. However, due to the importance of ease in clinical application and the desire to enable a large number of practitioners to apply regenerative endodontic techniques, most current studies focus on understanding and developing cell‐homing strategies. This approach aims to attract endogenous cells, avoiding the complexity and cost associated with cell transplantation (Eramo et al. 2018), which is why it was chosen for this study.

Large animals have been widely used in regenerative endodontic experiments and studies, as they present clinical conditions that allow for the presence of functional teeth. Dogs, in particular, have been used in numerous tissue regeneration studies due to their strong similarities with humans in terms of development, growth, general organ function, pathology, and the anatomy and function of teeth. Additionally, stem cells derived from their dental pulp closely resemble those found in humans. For these reasons, dogs were chosen for this research (Pearce et al. 2007; Nakashima et al. 2019; Mangione et al. 2022).

A CG was included in which the teeth were left without interference to confirm the normal development of the tooth apex in the studied animals and compare the findings histologically with the other groups (AlHowaish et al. 2022).

However, despite the widespread use of chitosan in various applications, it has some limitations, such as its low solubility in neutral environments, which weakens its ability to interact effectively. Additionally, its weak physical properties, including brittleness and stiffness, are caused by strong hydrogen bonds between its molecules (Hoff, Liu, and Bollag 1985).

Kim et al. (2009) highlighted that the application of chitosan alone did not yield favorable results in terms of cell adhesion, proliferation, and differentiation (Kim et al. 2009).

Therefore, modifications of chitosan have been pursued to improve its properties. However, not all chitosan derivatives have proven effective in dental pulp regeneration. For example, Palma et al. noted that the addition of hyaluronic acid or pectin to chitosan did not enhance dental pulp regeneration in dogs' teeth (Palma et al. 2017).

Similarly, Ducret et al. found that although chitosan gel combined with fibrin provided some antimicrobial benefits, it did not show a clear improvement in cell adhesion and proliferation (Ducret et al. 2019).

However, enzymatic modification of chitosan has demonstrated distinct advantages, such as increased adhesion, proliferation, and differentiation of stem cells, along with enhanced antimicrobial efficacy. This was experimentally demonstrated by Aljawish and colleagues (Aljawish et al. 2014; Aljawish, Muniglia, and Chevalot 2016).

To date, no histological studies have been conducted on the application of enzymatically modified chitosan in pulp regeneration techniques or its comparison with other scaffolds. This necessitated the conduction of this study.

In this study, 2.5% NaOCl was used due to its effectiveness and low toxicity (Shah and Logani 2012; Brizuela et al. 2020), following the American Association of Endodontists (AAE) recommendations to maximize disinfection and support the survival of SCAP (AAE 2016).

Irrigation with 17% EDTA was then performed to induce the release of growth factors through surface demineralization of the dentin, enhancing MSC attachment and growth (Diogenes et al. 2013). Furthermore, the canals were thoroughly rinsed with a physiological saline solution to reduce potential direct toxicity on the stem cells (Mangione et al. 2022).

The scaffolds used in this study were applied in gel form due to their enhanced applicability, allowing for better access to the intricate and relatively small structures of the pulp canal, thereby facilitating improved cell migration (Chang et al. 2017).

Notably, both native chitosan and enzymatically modified chitosan scaffolds were combined into a BC to leverage the synergistic effect resulting from the combination of the scaffolds with the BC. This combination encourages the migration and adhesion of stem cells attracted to the pulp canal after the induction of bleeding from the apical end (Moreira et al. 2021).

MTA was selected as the capping material due to its widespread use in REPs in most of the published cases. Although newer barrier materials have been introduced, they are still undergoing evaluation for their effectiveness in REPs (Diogenes et al. 2013). MTA was positioned below the cementoenamel junction to prevent coronal staining (Chen et al. 2012), and this is to simulate the same conditions that can be applied to human teeth.

The results demonstrated that the formation of regenerated tissue was greater in the EMCS + BC group compared to the NCS + BC and BC groups. It also showed the formation of pulp‐like tissue in addition to the formation of odontoblast‐like cells in the EMCS + BC group.

This is attributed to the enzymatically modified chitosan's ability to enhance stem cell adhesion, proliferation, and differentiation. This was previously proven in the study of Aljawish et al. in 2016, who studied the growth of stem cells on chitosan films and compared them with enzymatically modified chitosan, and concluded that increasing the hydrophobic property and increasing surface roughness of the enzymatically modified chitosan increases cell adhesion and proliferation (Aljawish, Muniglia, and Chevalot 2016).

The regenerative tissue formation percentage in EMCS + BC and NCS + BC groups was higher than the BC group due to the synergistic effect resulting from combining the scaffolds with the BC. This hybrid scaffold stabilizes the blood clot, allowing cells to secrete an extracellular matrix rich in growth factors, the third important element in pulp regeneration, which increases cell differentiation (Staffoli et al. 2017).

Previous studies have highlighted the disadvantage of using a BC alone as a scaffold in pulp regeneration, noting its instability and rapid dissolution, which reduces its effectiveness in pulp regeneration using cell‐homing technology (Fehrenbach and Popowics 2019).

In the EMCS + BC group, the synergistic effect not only increased the number of stem cells and growth factors that help the differentiation of these cells but also, coupled with the enzymatically modified chitosan's ability to improve cell adhesion and proliferation, played a significant role in the appearance of odontoblast‐like cells in this group.

These results align with the results of Moreria et al. in a study in 2021, which demonstrated that applying native chitosan alone and without participating with the BC did not give optimal results in pulp regeneration compared to the hybrid scaffold that combines chitosan and a BC (Moreira et al. 2021). Similarly, the results were consistent with those of AlHowaish et al. (2022) and Londero et al. (2015), where the researchers showed that the combination of the scaffold and the BC led to the formation of dentin tissue along the canal walls.

However, the results of this study differed from those of Palma et al. who observed that the chitosan‐based scaffold application in combination with a BC did not improve mineralized tissue regeneration compared to the BC alone (Palma et al. 2017). This difference could be attributed to the possible variation in the properties of chitosan. Palma's study did not provide any details regarding the type of chitosan used, whereas our study used chitosan with a higher degree of deacetylation, which is known to enhance antimicrobial effectiveness (Andres et al. 2007). The results also differed from those of Abbas et al. who found that chitosan loaded with corticosteroids or demineralized bone scaffolds did not result in the formation of dentin or any dentin‐like cells (Abbas et al. 2020). This difference may also be due to the varying properties of the chitosan used, as Abbas used chitosan with a degree of acetylation greater than or equal to 40%.

The percentage of regenerating soft tissue (ST%) in the EMCS + BC group was greater than that in the BC group, with a statistically significant difference. This matter can be attributed to the ability of enzymatically modified chitosan to enhance the adhesion and proliferation of fibroblast cells, contributing to the formation of fiber‐rich connective tissue (Aljawish et al. 2014).

The regenerated tissue in our current study was vital connective pulp‐like tissue, with bone‐like tissue observed in some cases, but no cementum‐like tissue was noticed. The findings of this study are consistent with Arslan et al. (2019) who noted fibrous connective tissue rich in blood vessels with bone‐like tissue but no cementum‐like tissue (Arslan et al. 2019). They also align with AlHowaish et al. who reported the formation of connective pulp‐like tissue, rich in fibroblasts, fibers, and blood vessels (AlHowaish et al. 2022), However, the findings differed from those of Tawfik et al. who reported that the tissues formed in the root canals were cementum, bone, and periodontal tissue (Tawfik et al. 2013).

The limitations of this research include the challenge of isolating the teeth of the dogs due to the difficulty of finding suitable clamps. Excellent isolation was achieved using a surgical suction device and cotton rolls, especially since the dogs were under general anesthesia. Moreover, this research was not applied to human teeth that will give more accurate results. Immunological stains, which help identify the tissues and cells more accurately, were also not used. Furthermore, the study did not investigate the application of scaffolds in cases of teeth with periapical lesions to assess the potential of tissue regeneration in such situations.

Clinical and radiological studies are recommended to evaluate the application of enzymatically modified chitosan alone or in combination with a BC to assess its effectiveness in pulp regeneration.

## 5 Conclusion

In light of the limitations of this study, the following conclusions can be drawn:

- The application of enzymatically modified chitosan in combination with a BC as a scaffold for pulp regeneration, which was used for the first time in this research, gives excellent results. This facilitated the formation of pulp‐like tissue and odontoblast‐like cells along the dentin walls.
- The percentage of regeneration tissue formation in the enzymatically modified chitosan group was greater than that in the native chitosan group, and the formation of renewed tissue when both enzymatically modified chitosan and native chitosan were combined with a BC was greater than in the BC group alone.

## Author Contributions

Aliaa Alshahhoud conceptualized the idea, performed the treatment of dogs' teeth, and contributed to the writing, documentation, and interpretation of data; Nizar Issa provided the laboratory work for modifying the chitosan; Ahmad Manadili provided the histological study; Mhd. Salem Rikab and Nizar Issa conceptualized the idea and supervised the research; Yasser Alsayed Tolaibah contributed to the interpretation of data and the revision, formatting, and re‐editing of the manuscript. All authors have read and agreed to the published version of the manuscript.

## Ethics Statement

(1) Title of the Approved Project: A comparison between the application of three types of normal scaffolds on pulp regeneration. (2) Name of the Institutional Approval Committee or Unit: The Institutional Review Board of Damascus University—Faculty of Dentistry. (3) Approval Number: UDDS‐1189‐25092018/SRC‐74. (4) Date of Approval: September 25, 2018. Informed consent was obtained from all subjects/caregivers involved in the study.

## Conflicts of Interest

The authors declare no conflicts of interest.

## Data Availability

The data that support the findings of this study are available from the corresponding author upon reasonable request.
